# *Trypanosoma cruzi* 80 kDa prolyl oligopeptidase (Tc80) as a novel immunogen for Chagas disease vaccine

**DOI:** 10.1371/journal.pntd.0006384

**Published:** 2018-03-30

**Authors:** Augusto E. Bivona, Andrés Sánchez Alberti, Marina N. Matos, Natacha Cerny, Alejandro C. Cardoso, Celina Morales, Germán González, Silvia I. Cazorla, Emilio L. Malchiodi

**Affiliations:** 1 Universidad de Buenos Aires, Facultad de Farmacia y Bioquímica, Cátedra de Inmunología and Instituto de Estudios de la Inmunidad Humoral Ricardo A. Margni (IDEHU), UBA-CONICET, Buenos Aires, Argentina; 2 Universidad de Buenos Aires, Facultad de Medicina, Departamento de Microbiología, Parasitología e Inmunología and Instituto de Microbiología y Parasitología Médica (IMPaM), UBA-CONICET, Buenos Aires, Argentina; 3 Universidad de Buenos Aires, Facultad de Medicina, Departamento de Patología, Instituto de Fisiopatología Cardiovascular, Buenos Aires, Argentina; 4 Laboratorio de Inmunología, Centro de Referencia para Lactobacilos (CERELA-CONICET). Tucumán, Argentina; Instituto de Ciências Biológicas, Universidade Federal de Minas Gerais, BRAZIL

## Abstract

**Background:**

Chagas disease, also known as American Trypanosomiasis, is a chronic parasitic disease caused by the flagellated protozoan *Trypanosoma cruzi* that affects about 8 million people around the world where more than 25 million are at risk of contracting the infection. Despite of being endemic on 21 Latin-American countries, Chagas disease has become a global concern due to migratory movements. Unfortunately, available drugs for the treatment have several limitations and they are generally administered during the chronic phase of the infection, when its efficacy is considered controversial. Thus, prophylactic and/or therapeutic vaccines are emerging as interesting control alternatives. In this work, we proposed *Trypanosoma cruzi* 80 kDa prolyl oligopeptidase (Tc80) as a new antigen for vaccine development against Chagas disease.

**Methodology/Principal findings:**

In a murine model, we analyzed the immune response triggered by different immunization protocols based on Tc80 and evaluated their ability to confer protection against a challenge with the parasite. Immunized mice developed Tc80-specific antibodies which were able to carry out different functions such as: enzymatic inhibition, neutralization of parasite infection and complement-mediated lysis of trypomastigotes. Furthermore, vaccinated mice elicited strong cell-mediated immunity. Spleen cells from immunized mice proliferated and secreted Th1 cytokines (IL-2, IFN-γ and TNF-α) upon re-stimulation with rTc80. Moreover, we found Tc80-specific polyfunctional CD4 T cells, and cytotoxic T lymphocyte activity against one Tc80 MHC-I peptide.

Immunization protocols conferred protection against a *T*. *cruzi* lethal challenge. Immunized groups showed a decreased parasitemia and higher survival rate compared with non-immunized control mice. Moreover, during the chronic phase of the infection, immunized mice presented: lower levels of myopathy-linked enzymes, parasite burden, electrocardiographic disorders and inflammatory cells.

**Conclusions/Significance:**

Considering that an early control of parasite burden and tissue damage might contribute to avoid the progression towards symptomatic forms of chronic Chagas disease, the efficacy of Tc80-based vaccines make this molecule a promising immunogen for a mono or multicomponent vaccine against *T*. *cruzi* infection.

## Author summary

Chagas disease is a neglected tropical disease caused by the parasite *Trypanosoma cruzi* that affects more than 8 million people around the world. Unfortunately, the diagnosis is generally performed too late, when anti-parasitic drugs are no longer effective. About 30–40% of infected individuals progress toward a symptomatic stage with cardiomyopathy as the main manifestation, leading annually to approximately 12,000 deaths. Therefore, new strategies for the control of the parasite must be explored. In this context, prophylactic or therapeutic vaccination has arisen as an interesting alternative to control the disease. In the present work, we present a parasite virulence factor (*T*. *cruzi* 80 kDa prolyl oligopeptidase, Tc80) as a new antigen for vaccine development against Chagas disease. Tc80-immunized mice developed a strong specific humoral and cellular immune response and most importantly were protected against *Trypanosoma cruzi* infection. Upon *T*. *cruzi* challenge, vaccinated mice presented a reduced parasite burden and less indicators of tissue damage. These results make Tc80 an interesting candidate to develop a mono or multicomponent vaccine against Chagas disease.

## Introduction

Chagas disease, also known as American Trypanosomiasis, is a chronic parasitic disease caused by the flagellated protozoan *Trypanosoma cruzi*. It is estimated that 8 million people over the world are infected by this parasite and more than 25 million are at risk of contracting the infection. It is a vector-borne disease transmitted by insects from *Reduviidae* family (colloquially known as ‘kissing bugs’) but the parasite can also be transmitted by congenital route, blood transfusions, organs transplantation or by ingesting food contaminated with the infective stages of the parasite [1]. Despite of being endemic on 21 Latin-American countries, Chagas disease has become a global concern due to migratory movements [2].

*T*. *cruzi* infection presents two distinguishable stages. An usually asymptomatic acute phase, that lasts about 2 months and is characterized by a high level of parasites in blood. Unfortunately, the available parasiticide drugs for the treatment are only effective in this phase. On the other hand, the chronic phase begins when the parasitemia decreases. It can remain asymptomatic lifelong, however, about 30–40% of infected individuals develop heart or digestive manifestations. *T*. *cruzi* infection represents the main cause of infectious cardiomyopathy [3]. Different clinical trials were conducted in order to assess the etiological treatment on chronic phase and the outcomes were somehow discouraging. While the parasite burden was reduced, no improvement in heart manifestations was observed [4,5].

In this scenario, prophylactic vaccines have emerged as an interesting modality of disease control. In the current work, we propose the *T*. *cruzi* 80 kDa prolyl oligopeptidase (Tc80) as a promising vaccine candidate. Tc80 is an enzyme expressed in the extracellular blood trypomastigote and the replicative intracellular amastigote [6]. Tc80 is secreted by the parasite and it is able to degrade the major extracellular matrix components such as collagen and fibronectin, contributing to the invasion of the parasite to the mammalian cells [7]. Furthermore, it was demonstrated that selective inhibitors for Tc80 were able to block the parasite infection *in vitro* [6,8]. Considering these features, we evaluated Tc80 immunogenicity and its ability to confer protection against *T*. *cruzi* challenge in a murine model.

Three prophylactic vaccination strategies were carried out: 1) A sub-unit vaccine formulated with recombinant Tc80 (rTc80) plus oligodeoxynucleotides CpG (ODN-CpG) as adjuvant; 2) An attenuated bacterial vector delivering the Tc80 gene; and 3) A priming with Tc80 DNA delivered by a bacterial vector followed by a boosting with rTc80 + ODN-CpG (heterologous prime-boost strategy).

Here we demonstrate that Tc80-specific immune response elicited by the vaccination was able to confer protection during the acute and chronic phase of *T*. *cruzi* infection. These findings make Tc80 a promissory immunogen for a mono or multicomponent vaccine to prevent Chagas disease.

## Materials and methods

### Mice, parasites and cell lines

In this work, different groups of inbred mice from the BALB/c and C3H/HeN strains were used for immunization protocols. Mice breeding was carried out in the animal facilities of the “Instituto de Microbiología y Parasitología Médica” (IMPaM, UBA-CONICET). Experiments with animals were approved by the Review Board of Ethics of the School of Medicine (UBA, Argentina) and conducted in accordance with the guidelines established by the National Research Council [9]. Animal sample size was estimated by a power-based method [10].

*T*. *cruzi* bloodstream trypomastigotes (from RA, K98 or β-galactosidase- expressing Tulahuen strains) were obtained from blood of CF1 infected mice. Culture-derived epimastigotes were used to obtain the soluble fraction of a parasite lysate (F105) as described [11].

BHK-21 (ATCC CCL-10) cells were used to induce rTc80 expression on eukaryotic cells and Vero cells (ATCC CCL-81) were used for *T*. *cruzi in vitro* infection. Both cell lines were maintained in RPMI 1640 medium (Gibco) and routinely tested for *Mycoplasma spp*. infection with DAPI stain.

### Tc80 cloning, expression and purification

Tc80 gene was amplified by PCR from genomic DNA of *T*. *cruzi* strain RA as described [8]. For expression in bacteria, the forward primer (5’- GAGCCAT**ATG**CGCAGCGTTTACCC-3’) included *Nde*I restriction site (underlined) and the translation start codon (ATG, bold). Reverse primer (5’-GATACTCGAG**TCAGTGATGGTGATGGTGATG**CTCTTTCCACGAAGCATTGA-3’) included *Xho*I restriction site (underlined), translation stop codon (bold) and 6 his tag coding sequence (bold and underlined). Tc80 amplicon was cloned in the expression vector pET23a^+^ and the construct was used for transforming chemically competent *Escherichia coli* BL21 (DE3).

Recombinant Tc80 (rTc80) was induced for 18 h with 0.5 mM isopropyl-β-D-thiogalactopyranoside at 20°C and then purified in native conditions with a Ni^2+^-NTA-agarose resin. Purified rTc80 was dialyzed against phosphate-buffered saline (PBS) with 10% glycerol and LPS was removed with polimixin B-agarose (Sigma). Residual LPS was determined using HEK-Blue LPS Detection Kit (Invivogen) and it presented a concentration below the quantification limit: 0.3 ng/ml.

Additionally, for DNA vaccination, Tc80 gene was cloned in the eukaryotic plasmid pcDNA3.1^+^. In this case, the amplification was performed with the followings primers: forward primer: 5’-GAATAAGCTTGCCACC**A****TG**CGCAGCGTTTACCC-3’, including *Hind*III restriction site (underlined), the kozak sequence (double underlined) and the translation start codon (ATG, bold). The reverse primer: 5’-GCTCCTCGAG**TCA**CTCTTTCCACGAAGCATTGA-3’ contained a *Xho*I restriction site (underlined) and the translation stop codon (bold). Tc80-pcDNA3.1 plasmid was used for transforming electrocompetent bacteria *Salmonella enterica* serovar Typhimurium aroA SL7207 which were used as DNA-delivery system.

PCRs were performed with ZymoTaq DNA polymerase (Zymo Research) with an initial denaturation 95°C for 5 min, 30 cycles of denaturation (95°C, 1 min)–annealing (59.3°C, 1 min)–extension (72°C, 2 min) and a final extension 72°C, 10 min.

### Immunization protocols

Four groups of 7-week-old C3H/HeN (H2-K^k^ haplotype) mice (n = 5–6 animals per group) were immunized with four doses separated by ten days. Group I (GI): rTc80im, was immunized intramuscularly in the quadriceps muscles with 10 μg of rTc80 adjuvanted with 10 μg of ODN-CpG 1826 (Invivogen). GII: STc80, received 4 doses of attenuated *Salmonella* carrying a Tc80-coding eukaryotic plasmid via oral (1x10^9^ CFU/mouse). GIII: Prime boost group (Pboost) was a combination of the two previous groups. Animals received two doses of Tc80 DNA delivered by the attenuated *Salmonella* (1x10^9^ CFU/mouse) followed by two doses of 10 μg rTc80 + 10 μg CpG-ODN by intramuscular route. As control group (SaroA), mice were intramuscularly injected twice with PBS + 10 μg CpG-ODN and then two doses of attenuated *Salmonella* carrying an empty plasmid pcDNA3.1 by oral route [12].

### Enzyme-linked immunosorbent assay (ELISA) for determination of Tc80-specific antibody titers

Tc80-specific antibody titers (IgG, IgG_1_ and IgG_2a_) were determined as previously described [13]. Briefly, 96-well polyvinyl chloride plates (Nunc, Thermo Scientific) were coated with 0.2 μg rTc80/well for 1 h at 37°C. Then, non-specific binding sites were blocked with 3% BSA in PBS for 2 h at 37°C. Plates were washed three times with 0.05% Tween-20 in PBS and two-fold serial dilutions (in 1% BSA-PBS) of immunized or *T*. *cruzi* infected mice sera were added and incubated for overnight at 4°C. Next day, 3 washes were performed and anti-IgG-HRP (Sigma B6398) antibody (1/10000 dilution), or biotin-conjugated anti-IgG1 or anti-IgG2a antibody (Pharmingen Becton Dickinson) diluted 1/2000, were added as secondary antibodies and incubated for 1 h at 37°C. When biotinylated antibodies were used, an additional incubation with streptavidin-HRP (BD Biosciences) was carried out at 37°C for 30 min. Reactions were revealed with TMB (tetramethylbenzidine, BD OptEIA) and stopped with 4 N H_2_SO_4_. Absorbance at 450 nm was determined on an ELISA plate reader (Labsystems Multiscan EX). Antibody end-point titer was calculated as the reciprocal of the dilution with a DO_450nm_ = 0.5

### Evaluation of cell invasion inhibition by Tc80-specific antibodies

The ability of rTc80-specific antibodies to block parasite infection was assessed as previously described [14]. Briefly, *T*. *cruzi* blood trypomastigotes (Tulahuen strain) expressing *E*. *coli* β-galactosidase were pre-incubated with the sera of immunized mice (1/10 dilution) and subsequently used to infect Vero cells (5x10^4^ parasites/5x10^3^ cells). After an overnight incubation at 37°C, 5% CO_2,_ cells were washed with PBS to remove non-infecting trypomastigotes and the culture was maintained for 4 more days. Cells were lysed with Nonidet P40 (1% v/v) and β-galactosidase activity was spetrophotometrically measured against the chromogenic substrate chlorophenol red β-D-galactopyranoside (CPRG). Inhibition percentage was calculated as follows: {1 - [(ABS_595nm_ of cells infected with parasites in presence of immunized mice serum)/(ABS_595nm_ of cells infected with parasites in presence of non-immunized mice serum)]} x 100.

### Enzymatic activity inhibition assay

Recombinant Tc80 (4 nM) was pre-incubated with 1/10 dilutions of sera from immunized mice. Then, residual prolyl oligopeptidase activity was determined in the presence of the dipeptide Z-Gly-Pro-AMC (Bachem), the fluorogenic substrate. The formation of the 7-amino-4-methyl coumarin (AMC) product was monitored by fluorometry (λ_excitation_ = 355 nm and λ_emission_ = 460 nm). Reactions were carried out at 37°C in a final volume of 100 μl reaction buffer (25 mM Tris, 250 mM NaCl, 2.5 mM DTT, pH 7.5) in a 96-well black plate (Costar Corning). Fluorescence measurements were made on a PerkinElmer Victor^3^ fluorimeter.

The AMC formation was recorded over time in relative fluorescence units (RFU) and the slope (ΔRFU/Δtime) from the linear region of the curve was used for the calculation of initial reaction velocity (Vi).

### Intracellular cytokine staining

Splenocytes from immunized mice (1x10^6^/200 μL) were incubated in the presence or absence of rTc80 (10 μg/ml) with complete RPMI medium in 96-well U-bottom plates. After 4 h of incubation, brefeldin A was added at 10 μg/ml and 12 h later, surface and intracellular cytokine staining was carried out.

Splenocytes were incubated with an APC-labeled anti-mouse CD4 antibody (eBioscience Inc.) for 45 min at 4°C, fixed with 2% PFA for 15 min at RT and incubated for 45 min at 4°C in permeabilization buffer with PE-labeled anti-IFN-γ and PE-Cy7-labeled anti-TNF-α antibodies (eBioscience Inc).

To perform suitable data acquisition and analysis, autofluorescence, single-stained and FMO (fluorescence minus one) controls were included. Stained cells were passed through the BD FACSaria II flow cytometer.

### *Ex-vivo* cytotoxicity assay

Cytotoxic ability of splenocytes from immunized mice (effector cells) to induce death on cells loaded with a Tc80 peptide (target cells) was studied. For target cells preparation, spleen cells from non-immunized C3H/HeN mice were incubated in the presence of 10 μM Tc80 peptide SEAELRKKI (H-2K^k^) or a non-related peptide to Tc80 sequence (AEEAFRLSV) [15] for 4 h at 4°C. These cells were washed with PBS and stained with 5 μM CFSE (CellTrace CFSE, Invitrogen) as described by manufacturer. Effector cells (1x10^6^) obtained from immunized mice were co-incubated with 1x10^5^ target cells for 4 h at 37°C and 5% CO_2_. Finally, the cells were washed with PBS and stained with 5 μg/ml propidium iodide (PI) to assess target cell death by flow cytometry (CFSE^+^PI ^+^ cells).

The percentage of specific death was calculated with the following formula [16]:

%Specificdeath=[(%CFSE+PI+%CFSE+)Tc80peptideloaded(%CFSE+PI+%CFSE+)non–loaded−1]×100

### *In-vivo* cytotoxicity assay

Spleen cells from non-immunized C3H/HeN mice were incubated for 4 h at 4°C with Tc80 peptide (10 μM) and stained with 8 μM CFSE (CFSE_high_ population). Another group of splenocytes was loaded with a non-related peptide (AEEAFRLSV) and was stained with 3 μM CFSE (CFSE_low_ population). Equal amounts of both populations were mixed and 2x10^7^ cells were intravenously injected into immunized C3H/HeN mice in a volume of 100 μl. After 18 h, the splenectomy was performed, the spleens were disrupted and the splenocytes suspension was analyzed by flow cytometry (BD FACScanto).

The calculation of the specific lysis was done with the following formula[17]:

%Specificlysis=[1−(CFSEhighCFSElow)immunizedmice(CFSEhighCFSElow)controlmice(SaroA)]×100

### Lethal challenge with *T*. *cruzi*

Two weeks after last immunization, immunized female C3H/HeN mice were challenged intraperitoneally with 200 blood trypomastigotes of *T*. *cruzi* strain RA. Parasitemia was monitored by counting blood parasites every 2–3 days in a Neubauer chamber. For this purpose, a 1/5 dilution of blood in lysis buffer (0.75% NH4Cl, 0.2% Tris, pH 7.2) was made. Mice deaths were daily recorded.

### Vaccine efficacy in a sub-lethal challenge

Two weeks after the last dose of vaccination protocols, immunized male C3H/HeN mice were challenged intraperitoneally with 2.5x10^5^ blood trypomastigotes of the sub-lethal *T*. *cruzi* strain K98. Parasitemias were recorded weekly during the acute phase. At 100 days post-infection (dpi), different parameters of disease outcome were assessed.

### Serum levels of myopathy-associated enzymes

Target tissue damage was assessed at 100 dpi by determining serum activity of the creatine kinase (CK) and its cardiac isoform (CK-MB), glutamate oxalacetate transaminase (GOT), and lactate dehydrogenase (LDH). Enzyme activity determinations were performed by spectrophotometry at 340 nm with commercial kits following the instructions given by the manufacturer (Wiener Lab). Results were expressed as absorbance variation per minute (ΔABS/min).

### Electrocardiograms (ECG)

Immunized and subsequently infected mice were anesthetized with ketamine-xylazine (100 mg ketamine and 16 mg xylazine/kg mouse) at 100 dpi and heart electrical activity was recorded with a Temis TM-300-V electrocardiograph. Corrected QT interval was calculated by the Bazett formula adapted for mouse [18].

### Histological analysis of target tissues

At 100 dpi, skeletal (quadriceps) and heart muscles from immunized and subsequently infected mice were dissected and fixed with 4% formalin in PBS. Then the material was embedded in paraffin, sectioned and stained with hematoxylin and eosin. Finally, 10 microscopic fields (100x magnification) were analyzed in 10 sections of each sample. Inflammation was qualitatively evaluated according to the number and spreading of inflammatory foci. Samples were classified with the following score: (+) isolated foci; (++) multiple non-confluent foci; (+++) multiple confluent foci; and (++++) multiple diffuse foci [19,20].

### Quantitative PCR (qPCR) for parasite burden

Parasite burden in skeletal (quadriceps) and heart muscle at 100 dpi was determined as described by Cummings et al. [21]. Briefly, total DNA was extracted from about 50–100 mg of muscle using a phenol-chloroform-isoamyl alcohol mixture (25:24:1 v/v, QuickDNA, Kalium Technologies). DNA concentration was adjusted to 25 ng/μL and it was used as template for DNA amplification with *T*. *cruzi*-specific primers (Pf: 5’- GGCGGATCGTTTTCGAG -3’, Pr: 5’- AAGCGGATAGTTCAGGG -3’). Samples were also amplified with mouse TNF-α-specific primers as normalizer gene (Pf: 5’- TCCCTCTCATCAGTTCTATGGCCCA -3’, Pr: 5’- CAGCAAGCATCTATGCACTTAGACCCC -3’). PCR reaction was performed using HOT FIREPol EvaGreen qPCR Mix Plus (Solis Biodyne).

To make the standard for parasite burden quantification, about 500 mg of muscle of non-infected mouse were mixed with 1x10^8^ *T*. *cruzi* epimastigotes. Total DNA was extracted and the concentration was also adjusted to 25 ng/μL. To construct standard curve, ten-fold serial dilution of the standard were made using non-infected mice muscle DNA (25 ng/μL) as diluent. Parasite burden was expressed as parasite equivalent/50 ng of total DNA.

### Statistical analysis

Statistical analysis was performed using 1-way ANOVA along with the post-tests indicated in each trial. The homogeneity of variances was validated using the Levene test. Normality was verified using the Shapiro-Wilks test. The log-rank test (Mantel-Cox) was used to analyze the survival curves using the Prism 6.0 program (GraphPad, San Diego, CA). The statistical analyses were referred to the control group of each experiment, except when indicated. Values of p < 0.05 were considered significant.

## Results

### Recombinant Tc80 expression

**Fig 1:**
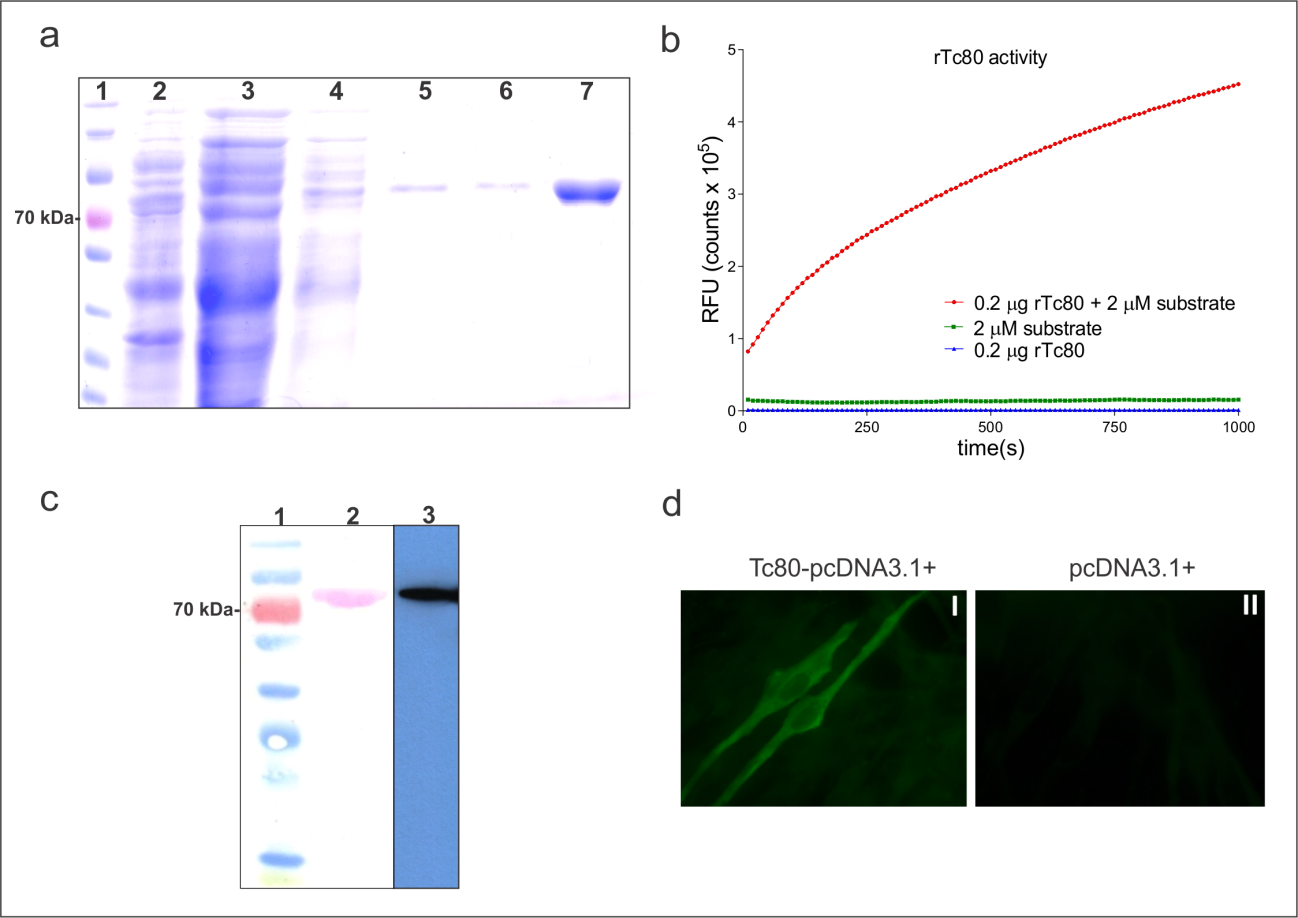
Recombinant Tc80 expression, purification and characterization. **a)** SDS-PAGE analysis of rTc80 expression and purification. Lane 1: MWM, lane 2: post-induction *E*. *coli* lysate, lane 3: Ni^++^-NTA column flow through, lane 4: column wash, lanes 5–7: column rTc80 elution steps. **b)** Prolyl oligopeptidase activity against the substrate Z-Gly-Pro-AMC measured as relative fluorescent units (RFU) in the presence (red circles) or absence (green squares) of rTc80. A control without substrate was included (blue triangles). **c)** Immunoblot detection of rTc80 (lane 2) and native Tc80 in an epimastigote *T*. *cruzi* lysate (lane 3). Protein were transferred to a PVDF membrane and subsequently incubated with mouse anti-rTc80 antibodies and a peroxidase-conjugated anti-mouse IgG antibody. Lane 1: pre-stained MWM, lane 2: Purified rTc80 revealed with 4-Cl naphthol and lane 3: Native Tc80 revealed with an ECL substrate. **d)** Indirect immunofluorescence on BHK-21 cells transfected with Tc80-pcDNA3.1^+^ construction (I) or empty pcDNA3.1^+^ (II). Cells were permeabilized and incubated with mouse anti-rTc80 antibodies and subsequently with FITC-conjugated anti mouse IgG antibody.

Tc80 gene was cloned in pET23a+ plasmid and the construct was used to transform *E*. *coli* BL21 (DE). Bacterial rTc80 expression was induced O.N. and the protein was purified with a Ni^2+^-NTA agarose resin (**Fig 1A**). As shown in **Fig 1B,** the recombinant protein showed prolyl oligopeptidase activity. The identity of the protein was confirmed by immunoblot, as rTc80-specific polyclonal antibody recognized the native protein in a parasite lysate (**Fig 1C**).

To obtain a DNA vaccine for expression in mammals, the Tc80 gene was also cloned in the eukaryotic expression vector pcDNA3.1^+^_._ After transfection of BHK21 cells with this construction, the recombinant protein was evidenced in an indirect immunofluorescence assay (**Fig 1D**).

### Immunization with rTc80 plus CpG ODNs elicited a potent humoral immune response

**Fig 2:**
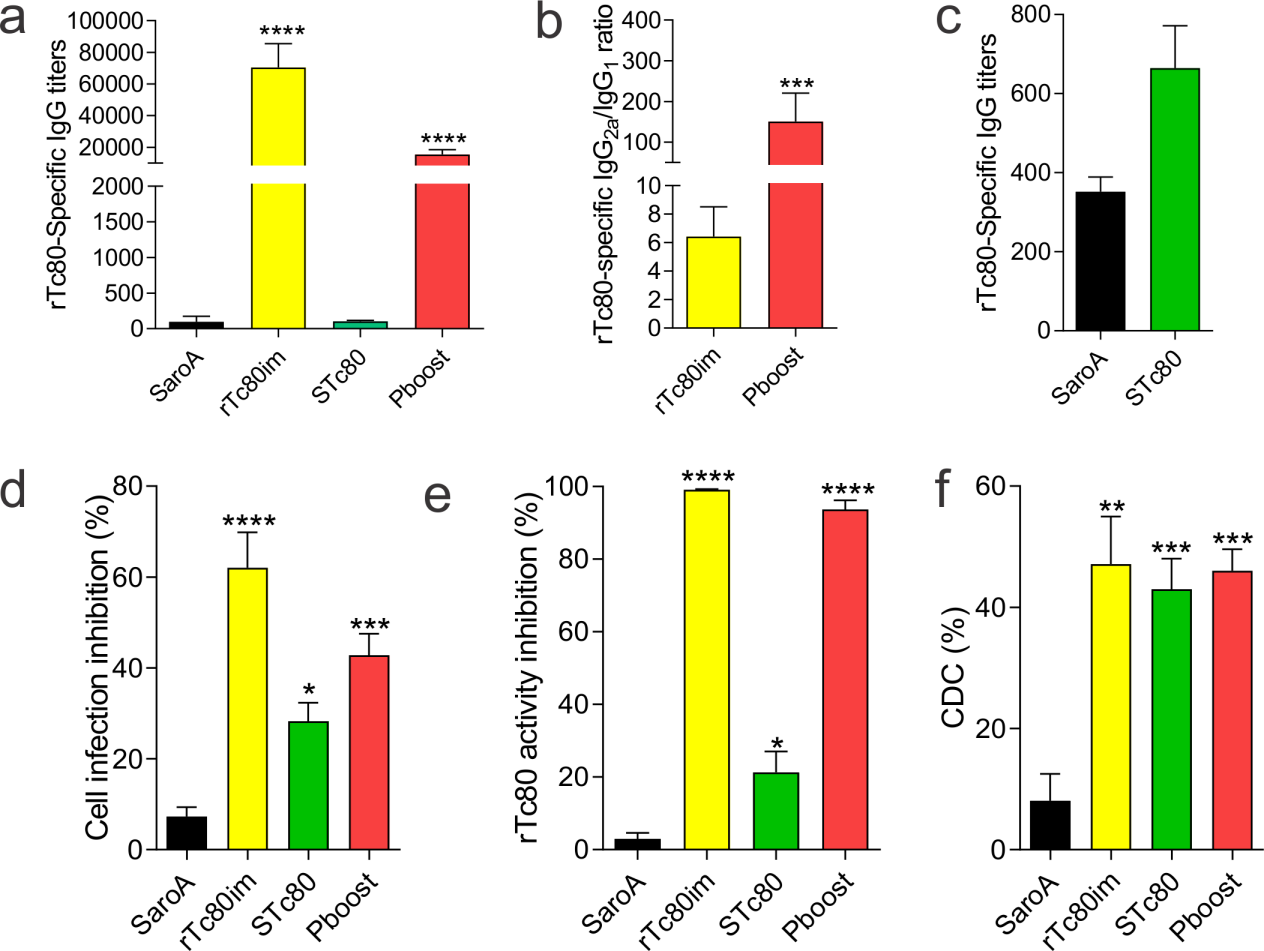
Humoral immune response and functionality of anti-rTc80 antibodies. **a)** ELISA for IgG anti-rTc80. Titer was calculated as the reciprocal of the dilution with a DO_450nm_ = 0.5. **b)** Tc80-specific IgG_2a_/IgG_1_ titer ratio determined by indirect ELISA using an isotype-specific secondary antibody. **c)** Biotin-Streptavidin amplification ELISA for rTc80-sepecific antibodies detection in STc80 group. **d)** Neutralization of *T*. *cruzi* non-phagocytic cell infection by sera from immunized mice. **e)** Prolyl oligopeptidase activity inhibition by rTc80-specific antibodies. **f)** Ability of Tc80-specific antibodies to mediate complement-dependent cytotoxicity (CDC). The percentage of lysis was calculated as {1 - [(No. of live parasites pre-incubated with sera from immunized mice)/(No. of live parasites pre-incubated with normal mouse serum)]} × 100. Results are expressed as mean ± SEM (n = 5–6 per group) and are representative of at least three independent experiments. *p<0.05; **p<0.01; ***p<0.001; ****p<0.0001. One-way ANOVA plus Dunnett’s post-test(a, d, e, f) and Student’s t-test (b, c).

To analyze the humoral immunity triggered by the immunization protocols, Tc80-specific antibody titers in serum were determined by ELISA at day 15 after last immunization dose. We observed that mice immunized at least twice with the recombinant protein (rTc80im and Pboost group) elicited antibody titers considerably higher than control group (SaroA) (p<0.001). By contrast, STc80 group which was immunized only with Tc80 DNA carried by *Salmonella*, did not elicit significant specific antibody titer comparing to SaroA (**Fig 2A**). Besides, antibodies isotypes reflected a Th1-biased response since IgG_2a_ levels were higher than IgG_1_ (**Fig 2B**). Interestingly, Pboost group showed an IgG2a/IgG1 ratio about 25-fold higher than rTc80im group, indicating that STc80 priming accentuated the bias towards a Th1 response.

Antibodies against rTc80 in STc80 group were only differentiated from control in an ELISA using a biotin-streptavidin signal amplification (**Fig 2C**). This observation is concordant with our previous experience in DNA immunization with attenuated *Salmonella* as carrier, that showed detectable, functional, but modest titers of specific antibodies [22,23].

### Antibodies anti-rTc80 mediated different functions for effective parasite control

As the antigen-specific B-cell response plays a role in protection [24,25], we assessed not only the magnitude but also the functionality of antibodies from immunized animals.

Neutralization of trypomastigotes cell infection was detected in sera from all vaccinated animals (**Fig 2D**). However, higher neutralization capacity was observed in rTc80im and Pboost groups that showed greater antibody titers. These groups were able to block nearly 50% of *T*. *cruzi* infection (**Fig 2D**). Moreover, anti-Tc80 antibodies from immunized animals were able to inhibit almost a 100% of the prolyl-oligopeptidase enzyme activity (**Fig 2E**), a fact that also correlated with the level of Tc80-specific antibodies.

Furthermore, antibodies from all the immunized mice were able to trigger trypomastigotes lysis in a complement-dependent cytotoxicity assay. Nearly 40% of parasite lysis was detected upon addition of a complement source independently of the vaccination protocol (**Fig 2F**).

### Immunization with STc80 triggered a strong antigen-specific cell-mediated immune response

**Fig 3:**
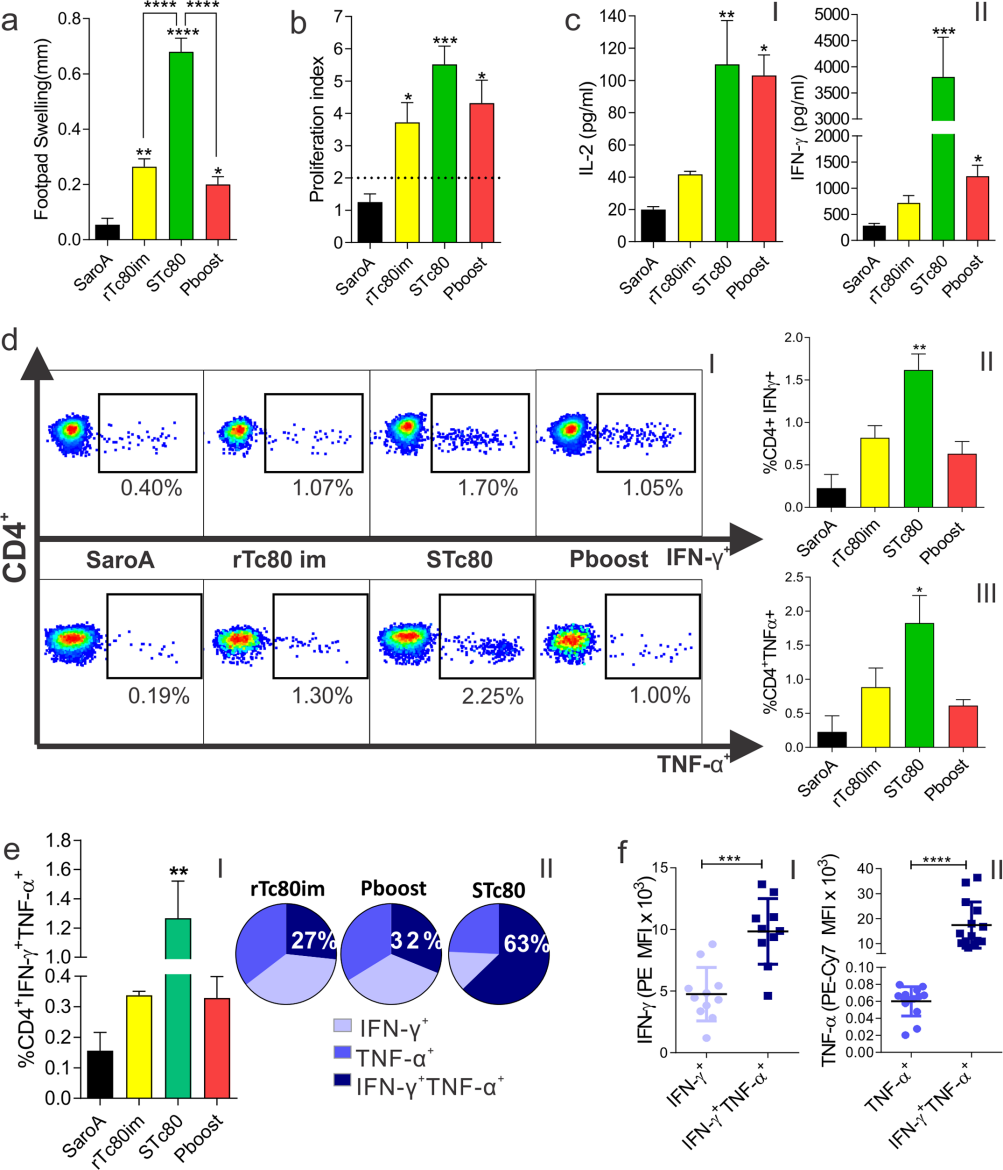
Overall cell-mediated immune response and CD4^+^ cells functionality in immunized mice. **a)** DTH reaction. Fifteen days after the last immunization, mice were subcutaneously injected in the hind footpad with rTc80. Footpad thickness was measured before and 48 h after rTc80 administration. The result was expressed as the difference between the two measurements. **b)** Antigen-specific proliferation. Splenocytes from immunized mice were stimulated with rTc80 and proliferation was assessed by 3H-thymidine uptake. Results were expressed as proliferation index: [cpm (counts per minute) in the presence of the antigen/cpm in the absence of the antigen]. **c)** Cytokines secretion by splenocytes from immunized mice upon antigen recall (I) IL-2 and (II) IFN-γ, determined by capture ELISA. **d)** Representative dot plots of intracellular cytokine production by CD4^+^ T cells. (I); Percentage of IFN-γ (II) or TNF-α (III) producing CD4^+^ cells. **e)** Analysis of CD4^+^ T cells polyfunctionality. I) Percentage of CD4^+^ T cells producing simultaneously IFN-γ and TNF-α. II) Proportion of CD4^+^ T cells with different degrees of functionality. **f)** Cytokine producing ability of mono and polyfunctional CD4^+^ cells for IFN-γ (I) and TNF-α (II), expressed as mean fluorescence intensity (MFI). Results are expressed as mean ± SEM (n = 5–6 per group) and are representative of at least three independent experiments. *p<0.05; **p<0.01; ***p<0.001; ****p<0.0001. One-way ANOVA plus Tukey’s post-test (a-e) and Student’s t-test (f).

To assess *in vivo* cell-mediated immunity, a delayed-type hypersensitivity (DTH) reaction was carried out on mice footpad. We found that all immunized groups developed a strong antigen-specific cellular response compared with SaroA control group. Among them, STc80 group showed the highest DTH reaction (**Fig 3A**). This result correlated with the proliferation of spleen cells that was evaluated *ex vivo* upon antigen re-stimulation where a similar pattern was obtained with STc80 group showing the highest response (**Fig 3B**).

Furthermore, splenocytes from all immunized mice were able to secrete IL-2 and IFN-γ upon antigen recall, being these differences significant in STc80 and Pboost compared with SaroA (**Fig 3C-I and II**).

### STc80 immunization stimulated the generation of polyfunctional CD4^+^ T cells

By flow cytometric analysis we found that all immunized groups presented a higher percentage of IFN-γ or TNF-α producing CD4^+^ T cells compared with SaroA group, though it was only significantly higher only in STc80 (**Fig 3D**). Moreover, the frequency of antigen-specific polyfunctional CD4^+^ cells that simultaneously produced both IFN-γ and TNF-α was significantly increased in STc80 group (**Fig 3E-I**), representing more than 60% percent of the cytokine-producing cells within this group (**Fig 3E-II**). These polyfunctional populations were also associated with a high extent of cytokine production compared with single cytokine-producing cells (**Fig 3F**). In that way, mean fluorescent intensity (MFI) of IFN-γ channel indicates that double positive CD4^+^ lymphocytes produced 2 times more IFN-γ compared to that of single positive cells. Similarly, a 300-fold increase was detected in the TNF-α production of these cells.

### Immunized mice showed CTL activity against target cells presenting a Tc80 epitope

In order to assess the immune response mediated by cytotoxic T lymphocytes (CTL), we searched for Tc80 H2-K^k^ epitopes with different algorithms: SYFPEITHI [26], RANKPEP [27–29] and IEDB [30]. From peptide prediction results (**S1 Dataset**) we selected and synthesized the one with the highest MHC-I binding score: SEAELRKKI.

**Fig 4:**
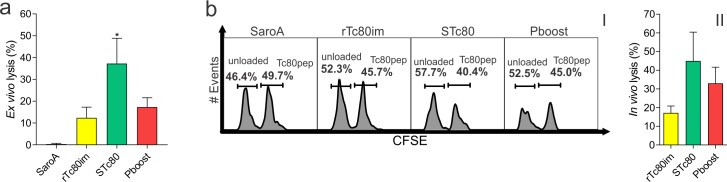
Tc80-specific CTL activity in immunized mice. **a)** *Ex vivo* CTL activity. Splenocytes from immunized mice were incubated with cells loaded with the SEAELRKKI peptide (target cells, CFSE^+^). Later on, cell death was assessed with propidium iodide by flow cytometry and lysis percentage was calculated according to the following formula: {[(%CFSE^+^PI^+^/CFSE^+^)_loaded_/(%CFSE^+^PI^+^/CFSE^+^)_non-loaded_]-1}*100. **b)** *In vivo* CTL activity. Nonapeptide-pulsed and CFSE_high_-labelled target cells were transferred to immunized mice and 16 h later, target cell lysis was evaluated in splenocytes by flow cytometry. Mice were also administered with non-loaded and CFSE_low_-labelled cells for non-specific cell lysis control. (I) Representative histograms gated on CFSE positive cells showing CFSE_low_ and CFSE_high_ populations and (II) bar chart expressing the percentage of lysis as {1-[(%CFSE_high_/%CFSE_low_)_immunized_/(%CFSE_high_/%CFSE_low_)_SaroA_]}*100. Results are expressed as mean ± SEM (n = 4–5 per group) and are representative of three independent experiments. *p<0.05. One-way ANOVA plus Dunnett’s post-test.

CTL activity against the predicted H-2K^k^ nonapeptide was then evaluated *ex vivo* and *in vivo*. In the *ex vivo* approach, splenocytes from immunized mice induced death of target cells loaded with Tc80 peptide. We observed that the cytotoxic activity of STc80 group was significantly higher than the control group (**Fig 4A**). This result was further confirmed with an *in vivo* citotoxicity assay. Nonapeptide-pulsed and CFSE-labelled target cells were transferred to immunized mice and 16 h later, specific cell lysis was evaluated in the spleen by flow cytometry. Vaccinated mice presented higher *in vivo* CTL activity compared to control group since immunized animals showed a reduced frequency of peptide-pulsed cell population (CFSE_high_) with respect to the non-specific lysis subset (CFSE_low_) (**Fig 4B-I**). Similar to what we observed for the CD4^+^ population, STc80 group presented the highest level of CTL functionality between immunized animals (**Fig 4B-II**). Taken together, these results show that immunization with the Tc80 antigen was able to elicited specific CTL response that may contribute to the elimination of *T*. *cruzi* infected cells.

### Tc80 immunization conferred protection against a lethal *T*. *cruzi* challenge

**Fig 5:**
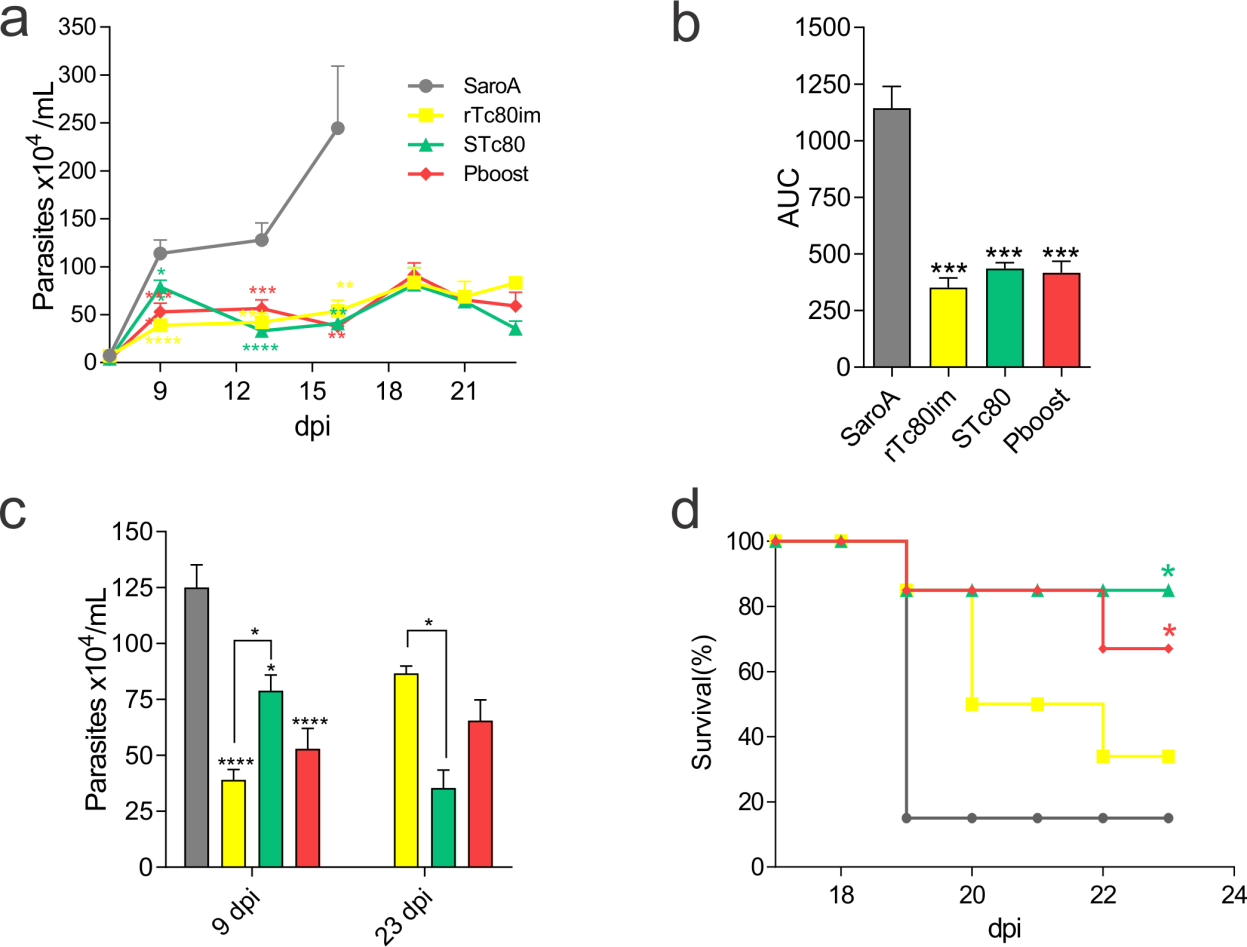
Protection against a lethal *T*. *cruzi* challenge. **a)** Parasitemia curve at different days post-infection (dpi) and b) area under the curves (AUC) of parasitemia. **c)** Parasitemia at early (9 dpi) and late (23 dpi) stages of acute infection. **d)** Survival rate curve. Results are expressed as mean ± SEM (n = 4–5 per group) and are representative of at least three independent experiments. *p<0.05; **p<0.01; ***p<0.001; ****p<0.0001. One-way ANOVA plus Dunnett’s post-test(a, b) or Tukey’s post-test (c) and log-rank test for survival curves (d). Color code from panel ‘a’ legend is also used in the other panels (b, c, d).

To evaluate if the immunization protocols were able to confer protection, vaccinated mice were challenged with the highly virulent *T*. *cruzi* RA strain. All immunized mice presented significantly reduced parasitemias during the acute phase of infection compared to the control (**Fig 5A**) and this was reflected in a 3-fold reduction of the areas under the curve (**Fig 5B**). We observed that on the early acute phase (9 dpi) rTc80im group achieved the highest control of parasitemia. By contrast, at 23 dpi when most of the control animals died, parasitemias were significantly lower in STc80 and Pboost compared with rTc80im (**Fig 5C**).

More important, all vaccinated mice had an increased in the survival rate compared with SaroA group. Specifically, those mice immunized with at least 2 doses of *Salmonella sp*. carrying the Tc80 gene presented a higher survival rate at 23 dpi: 80% and 67% for STc80 and Pboost, respectively (**Fig 5D**).

### Tc80-based vaccine prevented chronic disease-associated damages

**Fig 6:**
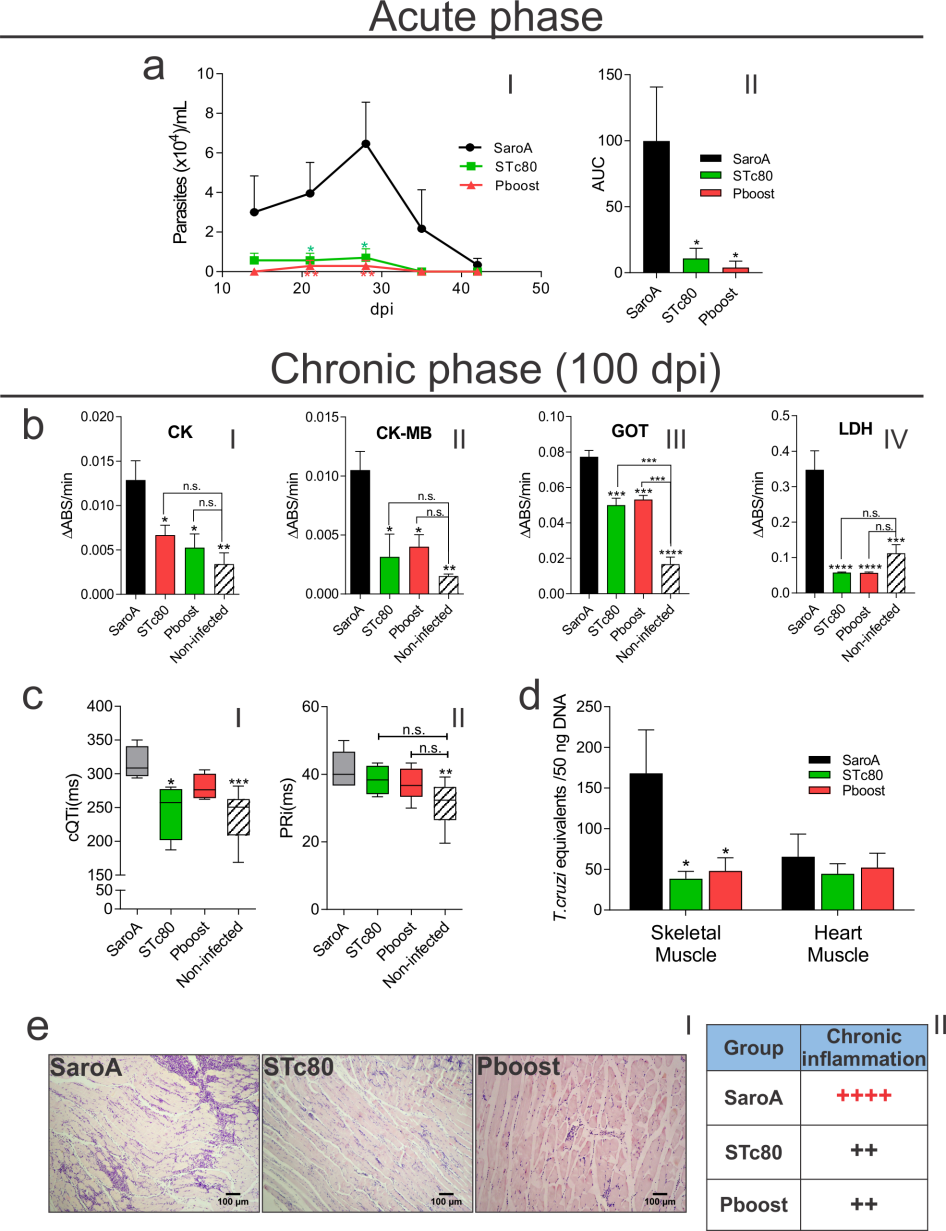
Protection in a chronic infection model in vaccinated mice. Animals were immunized as indicated and challenged 15 days later with *T*. *cruzi* K98 strain. **a)** Parasitemia curve (I) and its AUC (II) during the acute phase of infection. **b)** Serum level of tissue damage-associated enzymes at 100 dpi: creatine kinase, CK (I) and its cardiac isoform, CK-MB(II); glutamate oxaloacetate transaminase, GOT (III); and lactate dehydrogenase, LDH (IV). **c)** Electrocardiogram parameters at 100 dpi: Corrected QT interval(cQTi) (I) and PR interval(PRi) (II). **d)** Parasite load by qPCR in target tissues at 100 dpi. **e)** Histopathological analysis of skeletal and heart muscle at 100 dpi. Representative muscle sections stained with hematoxilin-eosin at 100x magnification (I) and semi-quantitative analysis of inflammatory infiltrate (II). Results are expressed as mean ± SEM (n = 4–5 per group) and are representative of two independent experiments. Survival statistical analysis was performed with log-rank test. *p<0.05; **p<0.01; ***p<0.001; ****p<0.0001. One-way ANOVA plus Tukey’s post-test. (n.s.: non-significant differences).

As *T*. *cruzi* is able to persist, even in vaccinated animals, the ability of the Tc80-based vaccine to prevent chronic phase-associated disorders, was evaluated after a challenge with the sub-lethal *T*. *cruzi* K98 strain (K98). In accordance with the observations after the lethal challenge, immunized mice presented lower parasitemias with respect to SaroA group during the acute phase of infection (**Fig 6A**). Since rTc80im group was not significantly protected against *T*. *cruzi* lethal challenge, this group was not subjected to a K98 infection.

At the chronic stage (100 dpi), immunized mice showed significantly lower serum levels of tissue damage-associated enzymes with respect to control group, more importantly these values were similar to those observed in non-infected mice (**Fig 6B**). Notably, immunization with Tc80 was able to avoid heart electrophysiological disorders such as corrected QT and PR intervals prolongation (**Fig 6C**). Thus, STc80-immunized and subsequently infected mice presented a QT interval significantly less prolonged than SaroA group and more interestingly they were similar to those registered in non-infected control mice (**Fig 6C-I**). Similar results were observed for the PR interval which tended to be less prolonged compared to control group and similar to non-infected mice (**Fig 6C-II**).

When qPCR was conducted on tissue samples, both immunized groups showed a 4-fold reduced parasite burden in skeletal muscle compared with SaroA group (**Fig 6D**). Additionally, by hematoxilin-eosine stain, we observed that immunized and infected mice also presented reduced extent of inflammatory infiltrates (**Fig 6E**). While SaroA group showed multiple diffuse inflammatory foci (++++), STc80 and Pboost groups showed non-confluent foci (++). On the contrary, in heart muscle, no significant difference was observed between the different groups. Interestingly, we found that parasite burden was relatively low (**Fig 6D**) which correlated with the low levels of isolated inflammatory foci observed in the histopathologic studies. Amastigotes nest in immunized and control groups were not detected neither in heart nor skeletal muscle.

## Discussion

Tc80 is a *T*. *cruzi* virulence factor involved in extracellular matrix degradation favoring tissue and cell invasion by the parasite. In addition, it has been shown that the inhibition of Tc80 enzymatic activity blocks non-phagocytic cell invasion [6,8]. In this scenario, we hypothesized that Tc80 would be an interesting target for the design of novel vaccines against Chagas disease.

To test Tc80 immunogenicity and its protective efficacy, we cloned and expressed it as a recombinant protein (rTc80). Tc80 cloned from *T*. *cruzi* RA strain presented 98–99% sequence identity with *T*. *cruzi* strains from different DTUs, including CL Brener, Tulahuen, SylvioX10/1 and Dm28c (GenBank Accession Numbers: XP_820337.1, AAQ04681.1, EKG04331, respectively). This high homology among different strains point out Tc80 as a good universal candidate for a vaccine against *T*. *cruzi* infection.

We were able to express soluble Tc80 with a high yield, and more importantly, the protein was enzymatically active against its specific substrate. This fact is relevant because it clearly indicates that Tc80 is adequately folded and therefore conformational epitopes would be available for antibody generation in a Tc80 based immunization.

Throughout this work we have used different vaccination protocols against *T*. *cruzi* infection in order to achieve protection. Thus, we explored a subunit vaccine constituted by rTc80, adjuvanted by ODN-CpG and administered intramuscularly. This adjuvant is a good inducer of humoral and cellular immune response with a Th1 profile [31] and have been tested with other *T*. *cruzi* antigens [13,16,32]. Considering the relevance of the cellular immune response to the control of *T*. *cruzi* infection, we also evaluated a vaccine based on Tc80 DNA [33]. To immunize with Tc80-coding DNA, we used an attenuated bacterial vector (*Salmonella enterica serovar Typhimurium aro*A SL7207) which was administered by oral route. This approach, among other advantages, increases transgene transfection efficiency compared with naked plasmid, has adjuvant effect due to the PAMPs from the attenuated bacteria and also presents a strong capacity to stimulate CTL and Th1 cell-mediated immune response, crucial for parasite control [25,34]. Our laboratory has previously demonstrated the efficacy of this DNA delivery system as a vehicle for different *T*. *cruzi* immunogens: Cruzipain [35,36], Tc52 [15] and Tc24 [36].

To combine the advantage of the above-mentioned formulations, a heterologous prime boost scheme was performed based on DNA priming and recombinant protein boosting. In this way, we aimed to join the strong cellular response triggered by DNA immunization and the powerful humoral response generated by recombinant protein immunization [37,38]. Different heterologous prime boost protocols (DNA + protein) have been implemented with different *T*. *cruzi* immunogens obtaining promising results; among them: Cz [39], TcG2 and TcG4 [40], Tc52 [41], etc.

In the present manuscript, we demonstrated that immunization protocols that included the recombinant protein (rTc80im and Pboost groups) elicited high titers of Tc80-specific IgG. In contrast, antibodies titers were low in the group immunized only with DNA carried by *Salmonella* (STc80).

As expected, in Pboost group, rTc80 boosting increased humoral response compared with STc80 DNA vaccine, and displayed an IgG_2a_ predominating isotype suggesting a Th1-oriented immune response. Although rTc80im group presented an important IgG_2a_/IgG_1_ ratio (~6), the Th1 response bias was much higher in a Pboost vaccinated mice since the IgG_2a_/IgG_1_ ratio was about 150. The same phenomenon has been described for other DNA-protein prime boost schemes against different intracellular pathogens such as *T*. *cruzi* [41], *Leishmania donovani* [42], *Mycobacterium tuberculosis* [43] and *Brucella spp*. [44]. This Th1-bias generated by DNA-priming could be antigen-specific due to the immune response elicited by Tc80 DNA, as well as a non-specific adjuvant effect of *Salmonella* delivery system.

Tc80-specific antibodies elicited by immunization were able to block parasite infection to non-phagocytic cells, mediate blood trypomastigote lysis by complement activation, and inhibit the enzymatic activity of Tc80. The latter effect might be directly related to the blocking of Tc80 activity as the use of specific enzyme inhibitors blocked the infection of non-phagocytic cells [6,8]. Interestingly, despite experimental infection in mice certainly induces Tc80-specific antibodies, we could not detect antibodies able to inhibit rTc80 enzymatic activity, even during the chronic phase of the infection (**S1 Fig**). All these results suggest that the elicited antibodies by vaccination would be effective in inhibiting parasite invasion to cells. Surprisingly, sera from STc80 group were able to mediate these functions despite their low specific antibody levels. In this regard, further exploration of STc80 sera with an amplification ELISA allowed us to evidence the presence of anti-Tc80 antibodies, although there was not significant difference compared to the control.

We did not found correlation between anti-Tc80 antibodies level and sera ability to lysis parasites by complement activation since the lysis rate was approximately 40%, in all vaccinated mice despite the differences in antibody titers between groups. Classical complement activation suggests that Tc80 is located at least temporarily in the parasite membrane. Although a vesicular location near the flagellar pocket has been described in trypomastigotes [6], its location on the surface cannot be ruled out since many *T*. *cruzi* protein has been shown to be temporary located on the membrane [45–47].

With regard to the cell-mediated immune responses triggered by the immunization protocols, we observed that all immunized groups developed a Tc80-specific cellular response, which was reflected in a high delayed hypersensitivity reaction, antigen-specific proliferation and production of Th1 cytokines (IL-2, IFN-γ and TNF-α) that are crucial for parasite control [48,49]. Additionally, we found that the administration of 4 doses of STc80 maximize the robustness of cell-mediated immunity. Surprisingly, while prime boost strategy was aimed to improve the immune response, halving the number of doses of attenuated *Salmonella* was noticeably detrimental in to the stimulation of cellular response. Similarly, halving the number of doses of recombinant protein affected the specific humoral response.

Polyfunctional CD4^+^ T cells are those that simultaneously perform at least 2 functions and generally present a protective correlate against different chronic infections such as HIV infection [50], hepatitis C [51], tuberculosis [52] and leishmaniasis [53]. We found that mice from STc80 group presented a significantly higher proportion of multifunctional T lymphocytes producing simultaneously IFN-γ and TNF-α.

Some authors described the loss of polyfunctionality of CD4^+^ T cells during chronic infections as an exhaustion phenomenon, similar to the very well characterized effect for the CD8^+^ subset [54]. This situation has also been described for *T*. *cruzi* human infections [55]. In this regard, Albareda et al. [56], described that patients with early *T*. *cruzi* infection preserve CD4 polyfunctionality (IFN-γ^+^ and TNF-α^+^), while individuals with longstanding infections have higher frequency of monofunctional cells (i.e. IFN-γ^+^), indicating that a lack of polyfunctionality is associated to Chagas disease progression. Thus, the fact that immunization with STc80 specifically induced an increase in the number of CD4^+^ IFN-γ^+^ TNF-α^+^ T cells becomes more relevant. In addition, we also demonstrated that these polyfunctional cells have a significantly higher cytokine production ability for each cytokine than monofunctional cells.

We also showed *in vitro* and *in vivo* that Tc80-based immunization protocols triggered CTL activity. We identified the SEAELRKKI as a MHC-I–restricted cytotoxic T cell epitope in the Tc80 antigen. Splenocytes from immunized mice were able to induce death of target cells loaded with this Tc80 H-2K^k^ epitope. These results highlight the importance of immunization with DNA to promote antigenic presentation by MHC-I and the subsequent activation of CD8^+^ T cells, which play an important role in eliminating *T*. *cruzi* infected cells [57]. Although we focus on the cytotoxic activity, other CD8^+^ cells functions such as the cytokine production and degranulation capacity remain to be studied in depth. It is probably that the increased secretion of cytokines by splenocytes from STc80 and Pboost groups with respect to rTc80im group has its origin in CD8^+^ T cells. Further studies will be focused on a broader peptide screening to identify other potential epitopes capable of being presented in the context of MHC-I.

To analyze whether the triggered immune response was able to confer protection in the acute and chronic phase of infection, different challenges were performed. In a lethal-acute infection model, vaccinated mice were challenged with *T*. *cruzi* RA strain. This strain is highly virulent, pantropic [58] and belongs to discrete typing unit (DTU) Tc VI [59]. We observed that all immunized groups presented lower parasitemias compared with the control group. However, only those mice that received at least 2 doses of *Salmonella* carrying Tc80 gene (STc80 and Pboost groups) showed a significantly higher survival with respect to the controls. The protection achieved in these groups highlights the crucial role of the stimulated cell-mediated immunity in controlling *T*. *cruzi* infection.

With the aim of resembling a human infection where most patients survive the acute phase [60], we implemented a non-lethal mice model of infection. This chronic model was carried out by infecting male C3H mice with *T*. *cruzi* K98 clone which is non-lethal, myotropic [58], and belongs to DTU TcI [59]. The rationale behind the choice of this strain was its low lethality allowing infected mice to survive the acute period and reach the chronic phase. Moreover, myotropism of K98 strain increases the probability for detecting muscle disorders. Additionally, in order to favor the development of chagasic pathology, we used male mice which are more susceptible to *T*. *cruzi* infection than female ones [61,62]. Once the challenge was performed, different parameters of tissue damage were evaluated at 100 dpi.

In experimental and natural *T*. *cruzi* infection, the damage of muscle fibers caused by parasitic persistence leads to increased blood levels of intracellular enzymes such as LDH, GOT and CK. Importantly, we observed that all immunized groups had lower serum levels of CK, CK-MB, GOT and LDH than the control group. In accordance with this, we showed that immunized mice presented less electrocardiographic alterations than those registered in the control group (SaroA). Prolongation of the QTc interval is one of the most frequently alteration described for *T*. *cruzi* murine infection model [63] and it is generally related to a slow ventricular repolarization [64]. We found that mice immunized with STc80 had a significantly less prolonged QTc interval (p<0.05) than control mice and most importantly, these values were similar to the QTc interval observed in non-infected mice.

Furthermore, the parasite burden in target tissues at 100 dpi was evaluated by qPCR. We observed lower parasitic loads in cardiac muscle compared with skeletal muscle. These results were consistent with the histopathological analysis where we found a few isolated inflammatory foci in heart muscle and a larger infiltrate in skeletal muscle of control infected mice. These differences between both target tissues may correspond to the K98 strain differential myotropism as well as differential immune response mounted on each tissue. Interestingly, we found that in the skeletal muscle of the immunized mice the parasite burden was significantly reduced compared with SaroA groups. These results are in agreement with the correlation between parasitism and inflammatory infiltrates as described in the literature [65–67]. Neither parasite burden nor inflammation was detected in heart tissue. In contrast, skeletal muscle, presented high parasitism and inflammation. Therefore, the ECG disorders and the elevated serum levels of CK-MB observed in SaroA group were not associated with an ongoing heart inflammatory process at 100 dpi. These physiological disorders may be the result of previous irreversible alterations despite the low inflammatory extent [68].

In conclusion, we have demonstrated that Tc80-based vaccines are able to confer protection against *T*. *cruzi* infection, and importantly this immunoprotection was extensive to different strains belonging to different DTUs. Ours results emphasize the importance of stimulating cell-mediated immunity by activation of polyfunctional CD4^+^ T cells and cytotoxic T lymphocytes in order to control *T*. *cruzi* infection. Even though our results highlight the importance of humoral responses as an effective weapon in the control of early stages of parasite infection, if cell-mediated immune response is not strongly stimulated by vaccination, mice are unable to survive against a lethal challenge, as we observed in rTc80im group.

Similar to other vaccine immunogens which were previously described for *T*. *cruzi*, formulations with Tc80 as DNA vaccine or in a prime boost strategy partially control the infection. Although the ideal vaccine will be the one that provides sterilizing immunity, avoiding progression to symptomatic forms of the chronic Chagas disease is indeed a very promising goal. Considering *T*. *cruzi* complexity, a multicomponent and/or chimeric vaccine approach has been proposed by us [36,69] and others [40,70]. In this context and based on the results presented here, Tc80 represents a novel target to be considered in the development of an effective vaccine against *T*. *cruzi* infection.

## Supporting information

S1 Dataset Tc80 MHC-I H-2K^k^ epitope prediction results. (PDF)

S1 Fig Tc80-specific humoral immune response during acute and chronic (90 dpi) *T*. *cruzi* infection. **a)** ELISA for IgG anti-rTc80. Ninety-six-well plates were coated with rTc80 and incubated with serial dilutions of mice sera (non-infected, *T*. *cruzi* acute-infected or 90 dpi chronic-infected). Biotin-Streptavidin amplification was used for antibodies detection and titer was calculated as the reciprocal of the dilution with a DO_450nm_ = 0.5. **b)** Immunoblot detection of Tc80-specific antibodies during *T*. *cruzi* infection. Recombinant Tc80 (lanes 1, 3 and 5) or the parasite lysate F105 fraction (lanes 2 and 4) were separated by SDS-PAGE, transferred to a nitrocellulose membrane and incubated with sera from acute-infected mice (lanes 1 and 2), chronic infected mice (lanes 3 and 4) or rTc80 immunized mice (lane 5). **c)** Prolyl oligopeptidase activity inhibition by sera from Tc80-immunized or *T*. *cruzi*-infected mice. Results are expressed as mean ± SEM (n = 5–6 per group) and are representative of at least three independent experiments. *p<0.05; ****p<0.0001. One-way ANOVA plus Dunnett’s post-test.
(PDF)
