# Supplementary material for: Trypanosoma cruzi 80 kDa prolyl oligopeptidase (Tc80) as a novel immunogen for Chagas disease vaccine
Source: PLoS Negl Trop Dis. 2018 Mar 30;12(3):e0006384. doi: 10.1371/journal.pntd.0006384 (PMC5895069; doi:10.1371/journal.pntd.0006384)
Supplement: S1 Dataset — (PDF) [file pntd.0006384.s001.pdf]

S1 Dataset

Recombinant Tc80 amino acid sequence

MRSVYPLARRSMAAYTMHNMTVPEPYDYLEDPENPETKTFVNEQNAFFEEYFASEAELRKKIFESISNSQDYPRTSN
PSYINGHYYYYHNSGLQNQSVLMRAMSLTDTAPSIFLDPNSMSSDGTTALKATAWSEDESMLAYSLSDKGSDWQR
IHVRRADTVEDTSDVIEWAKFTAIAWWHNLGFFYTRYPALQGDVDKGAETDAAQDAFICFHRIGRPQDEDVVILSV
PEHPQWNMGASVSDCHSYVIVVLFDGCEPHNLVWVAELPSVEKGLGSEPLVFKKLVNEFAGRYTYLGNEGSTFYFV
TTRDAPRKKIVSIDIHTGQETVIVEQQRSVLSQAALVKKTLLLAYLEDVKDVFYYCRLEDPTLNAIPLPIGTITSFFSDRK
KDFVSFKITSFLLPGRSFFLDINDPQSSLRVFKDDTVEGLLVDDFVTEQTFYNSSDGVRIPMFIVYRKGSVSSESPLLLY
GYGGFNIPLTPAFSSSRMVFLRDLGGVLAVLNIRGGGEYGEEWHDAGRRACKQNCFTDFIEGAKFLHRQGYGSPQ
TTAIMGGSNGGLLVAAVANQAPELFRCVVCRVGVLDMYKFHKFTIGHAWKSDYGDPEKEEDFRVLQQYSPLHNIK
SGIKYPAILVVTGDHDDRVVPLHSLKYVATLQHMNPNEGGPFLARIEVAAGHGAGKPTSKILREAGDIYTFIAKNINA
SWKEHHHHHH

Mouse MHC-I H-2K^k nonapeptide prediction results

Top 10 SYFPEITHI results (http://www.syfpeithi.de/bin/MHCServer.dll/EpitopePrediction.htm)

Pos	1 2 3 4 5 6 7 8 9	Score
54	S **E** A E L R K **K** I	25
362	L **E** D P T L N **A** I	22
419	D **D** T V E G L **L** V	22
487	R **D** L G G V L **A** V	22
168	I **E** W A K F T **A** I	21
220	D **E** D V V I L **S** V	21
202	T **D** A A Q D A **F** I	19
296	N **E** G S T F Y **F** V	19
307	R **D** A P R K K **I** V	19
255	C **E** P H N L V **W** V	18

Top 10 RANKPEP results (http://imed.med.ucm.es/Tools/rankpep.html)

RANK	POS.	N	SEQUENCE	C	MW (Da)	SCORE	% OPT.
1	54	YFA	**SEAELRKKI**	FES	1055.25	22.861	43.57%
2	32	LED	PENPETKTF	VNE	1044.13	20.669	39.39%
3	422	DDT	VEGLLVDDF	VTE	988.11	19.956	38.03%
4	166	DTS	DVIEWAKFT	AIA	1067.24	12.933	24.65%
5	440	YNS	SDGVRIPMF	IVY	1003.19	12.468	23.76%
6	592	WKS	DYGDPEKEE	DFR	1063.06	11.921	22.72%
7	683	AGD	IYTFIAKNI	NAS	1064.29	10.947	20.86%
8	575	VGV	LDMYKFHKF	TIG	1210.46	10.705	20.40%
9	207	AAQ	DAFICFHRI	GRP	1103.32	10.57	20.14%
10	679	ILR	EAGDIYTFI	AKN	1010.12	10.466	19.95%

Top 10 IEDB results (http://tools.immuneepitope.org/mhci/)

allele	start	end	length	peptide	percentile_rank
H-2-Kk	54	62	9	**SEAELRKKI**	0.1
H-2-Kk	168	176	9	IEWAKFTAI	0.2
H-2-Kk	296	304	9	NEGSTFYFV	0.3
H-2-Kk	202	210	9	TDAAQDAFI	0.6
H-2-Kk	275	283	9	SEPLVFKKL	0.6
H-2-Kk	362	370	9	LEDPTLNAI	0.6
H-2-Kk	42	50	9	NEQNAFFEE	1.1
H-2-Kk	458	466	9	SESPLLLYG	1.2
H-2-Kk	432	440	9	TEQTFYNSS	1.6
