# Supplementary material for: Trypanosoma cruzi 80 kDa prolyl oligopeptidase (Tc80) as a novel immunogen for Chagas disease vaccine
Source: PLoS Negl Trop Dis. 2018 Mar 30;12(3):e0006384. doi: 10.1371/journal.pntd.0006384 (PMC5895069; doi:10.1371/journal.pntd.0006384)
Supplement: S1 Fig — a) ELISA for IgG anti-rTc80. Ninety-six-well plates were coated with rTc80 and incubated with serial dilutions of mice sera (non-infected, T. cruzi acute-infected or 90 dpi chronic-infected). Biotin-Streptavidin amplification was used for antibodies detection and titer was calculated as the reciprocal of the dilution with a DO450nm = 0.5. b) Immunoblot detection of Tc80-specific antibodies during T. cruzi infection. Recombinant Tc80 (lanes 1, 3 and 5) or the parasite lysate F105 fraction (lanes 2 and 4) were separated by SDS-PAGE, transferred to a nitrocellulose membrane and incubated with sera from acute-infected mice (lanes 1 and 2), chronic infected mice (lanes 3 and 4) or rTc80 immunized mice (lane 5). c) Prolyl oligopeptidase activity inhibition by sera from Tc80-immunized or T. cruzi-infected mice. Results are expressed as mean ± SEM (n = 5–6 per group) and are representative of at least three independent experiments. *p<0.05; ****p<0.0001. One-way ANOVA plus Dunnett’s post-test. (PDF) [file pntd.0006384.s002.pdf]

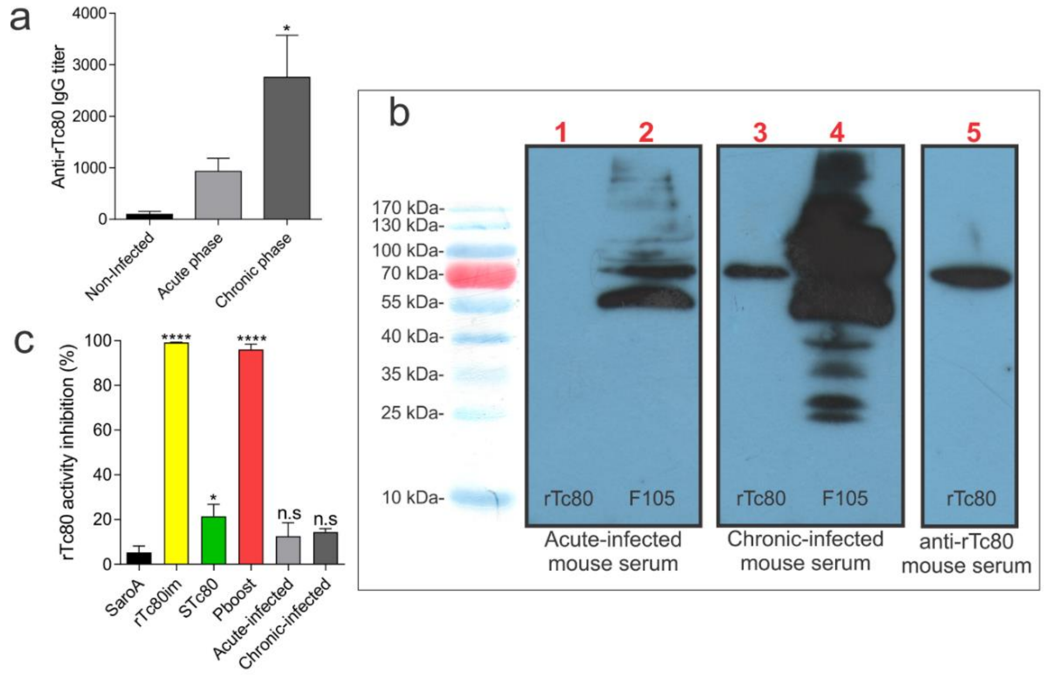

S1 Fig. Tc80-specific humoral immune response during acute and chronic (90 dpi) *T. cruzi* infection. a) ELISA for IgG anti-rTc80. Ninety-six-well plates were coated with rTc80 and incubated with serial dilutions of mice sera (non-infected, *T. cruzi* acute-infected or 90 dpi chronic-infected). Biotin-Streptavidin amplification was used for antibodies detection and titer was calculated as the reciprocal of the dilution with a DO_{450nm} = 0.5. **b)** Immunoblot detection of Tc80-specific antibodies during *T. cruzi* infection. Recombinant Tc80 (lanes 1, 3 and 5) or the parasite lysate F105 fraction (lanes 2 and 4) were separated by SDS-PAGE, transferred to a nitrocellulose membrane and incubated with sera from acute-infected mice (lanes 1 and 2), chronic infected mice (lanes 3 and 4) or rTc80 immunized mice (lane 5). **c)** Prolyl oligopeptidase activity inhibition by sera from Tc80-immunized or *T. cruzi*-infected mice. Results are expressed as mean ± SEM (n=5-6 per group) and are representative of at least three independent experiments. *p<0.05; ****p<0.0001. One-way ANOVA plus Dunnett's post-test.
